# Eda haplotypes in three-spined stickleback are associated with variation in immune gene expression

**DOI:** 10.1038/srep42677

**Published:** 2017-02-14

**Authors:** Shaun Robertson, Janette E. Bradley, Andrew D. C. MacColl

**Affiliations:** 1 School of Life Sciences, University of Nottingham, University Park, Nottingham, NG7 2RD, UK

## Abstract

Haplotypes underlying local adaptation and speciation are predicted to have numerous phenotypic effects, but few genes involved have been identified, with much work to date concentrating on visible, morphological, phenotypes. The link between genes controlling these adaptive morphological phenotypes and the immune system has seldom been investigated, even though changes in the immune system could have profound adaptive consequences. The *Eda* gene in three-spined stickleback is one of the best studied major adaptation genes; it directly controls bony plate architecture and has been associated with additional aspects of adaptation to freshwater. Here, we exposed F2 hybrids, used to separate *Eda* genotype from genetic background, to contrasting conditions in semi-natural enclosures. We demonstrate an association between the *Eda* haplotype block and the expression pattern of key immune system genes. Furthermore, low plated fish grew less and experienced higher burdens of a common ectoparasite with fitness consequences. Little is currently known about the role of the immune system in facilitating adaptation to novel environments, but this study provides an indication of its potential importance.

Adaptation to novel environments is an important cause of speciation^1^,^2^. Traditionally classified as allo- or sympatric, it is now clear that the geography of speciation is a continuum, and that divergence between populations often occurs with gene flow. In such conditions, theory predicts that selection should favour the concentration of adaptive phenotypic effects in a small number of genomic loci, in the form of pleiotropic genes or tightly physically linked haplotypes (‘supergenes’), with divergent alleles of major and diverse effects becoming fixed in different environments^3^,^4^,^5^. As yet there are few good examples of such loci, and the range of phenotypes that they can effect is poorly understood despite their potential contribution to speciation^6^. Given the ubiquity of parasites and pathogens in natural populations, it is reasonable to expect loci controlling divergence in immune system function to occur in local adaptation supergenes, yet evidence of such a link is lacking.

Although something of a ‘Holy Grail’ of evolutionary genetics, it has proven difficult to unequivocally identify the causal genetic variations underlying adaptive phenotypic variation and this necessarily hampers a complete understanding of the full range of phenotypic effects of such variants. Big strides have been made in this enterprise in recent years, particularly with the advent of modern sequencing technology. A number of examples now exist across a range of taxa where the region or gene underlying phenotypic variation has been identified, including the *Agouti* locus for colour polymorphisms in deer mice^7^,^8^, the *RXFP2* gene for horn architecture in Soay sheep^9^,^10^, the *Optix* gene controlling wing pattern in *Heliconious* butterflies^11^, two genes controlling petal colour morphs in annual phlox^12^, and the *Pomc* gene controlling melanin based colouration in a range of vertebrates^13^. The full range of potential adaptive consequences of any of these genes, however, is not yet understood.

One of the best documented examples of a locus that contributes to adaptation to new environments is *Ectodysplasin (Eda*) in the three-spined stickleback (hereafter ‘stickleback’), a small teleost fish found throughout the northern hemisphere which has repeatedly colonised freshwater from the sea. The *Eda* gene appears to be of fundamental importance in adaptation during this transition, and is located in a haplotype block that is associated with a diverse range of phenotypic effects^14^,^15^,^16^. It controls a polymorphism in lateral ectodermal bony (‘armour’) plates^17^, with two major alleles; the ancestral *Eda*^*C*^ allele (‘C’) and the derived *Eda*^*L*^ allele (‘L’). Across the Holarctic, marine populations carry the ancestral allele and have a full row of 32–36 plates, although the derived allele is present at a low frequency^17^. Many independently derived freshwater populations throughout the stickleback’s range are homozygous for the derived allele and are low plated, with less than 10 anterior plates only^18^,^19^,^20^. The *Eda* locus accounts for approximately 80% of observed variation in plate number^21^, with heterozygous individuals typically having an intermediate phenotype. Selection on *Eda* can be very strong^22^ and evolution can be extremely rapid^23^. Numerous selective agents have been suggested, with predation probably at least partly driving change^24^,^25^,^26^. Colonisation of freshwater, however, involves many other changes in environment and putative selective agents. Whilst there is strong selection on plate phenotype itself, the *Eda* gene experiences additional selection above this^27^. This may be the result of pleiotropic effects of *Eda* itself or other selective pressures on tightly physically linked genes^14^,^15^, of which parasites and the immune response may be one, although disentangling such patterns has proved difficult.

When stickleback colonise freshwater they experience major changes in the parasites that they encounter and consequently undergo rapid evolution of changes in parasite resistance^28^. There is increasing evidence of divergence in immune responses between locally adapted stickleback populations which appears to reflect the local parasite fauna present^29^,^30^,^31^. This is especially true of trematodes of the genus *Gyrodactylus*, which are common in saltwater but much less prevalent and abundant in freshwater^28^. We might thus expect selection to favour linkage between morphological and immunological adaptations to freshwater in the repeated evolution of stickleback to this major environmental transition^32^. However, whilst immune genes (especially those of the major histocompatibility complex) have been strongly implicated in stickleback local adaptation and speciation^29^,^33^,^34^, there is little evidence that immune genes are linked to those of other adaptive phenotypes in stickleback, or any other species (but see ref. 35). Suggestive evidence comes from the fact that several genes in tight linkage with *Eda* (referred to here as the *Eda* haplotype block) are major immune genes^17^. These include *Baff (Tnfsf13b*), a B cell activating factor^36^,^37^, *Garp*, which is involved in *Tgfβ* expression and regulation of T cell function^38^,^39^,^40^, and *Dusp1*, which plays a role in the control of inflammation^41^,^42^. The presence of genes with major roles in the immune system in tight linkage with *Eda* provides a potential mechanism by which the function of the immune system could change in concert with *Eda* during adaptation. In addition, *Eda* itself, as a member of the *Tnf* family, could also have such pleiotropic functions^43^,^44^. To date, however, there is no empirical evidence that the major *Eda* haplotype alleles have any association with immune function or parasite resistance in three-spined stickleback.

In this study, we created F2 hybrids between two populations with contrasting *Eda* genotypes (and thus plate phenotypes), and performed a common garden experiment in the field to examine the relationship between *Eda* genotype, parasite susceptibility and immune system function. We selected fish from two lake populations, a high plated saltwater resident population, Loch Ob nan Stearnain (‘Obse’, 57°36′9″N; 7°10′19″W), and a nearby low plated freshwater population, Loch à Chadha Ruaidh (‘Chru’, 57°35′38″; 7°11′51″W), on North Uist, Scotland. These populations show stable contrasts in parasite communities. Fish in Obse are typically infected with high levels of *Gyrodactylus arcuatus*, a monogenean ectoparasite that attaches to stickleback fins, skin and gills, but it is absent from Chru^28^. Fish in Chru show high levels of infection with the digenean trematode *Diplostomum gasterostei*, which penetrates the skin and migrates to the eye, but it is absent from Obse. Both of these parasites have fitness consequences for infected hosts^45^,^46^,^47^, and could therefore act as selective agents in their respective habitats.

By using F2 crosses, we randomised genetic background whilst creating fish with the three possible *Eda* genotypes, allowing us to focus on the *Eda* haplotype block whilst controlling for possible confounding effects of genetic background or maternal effects^48^,^49^. In this cross, *Eda*^*L*^ homozygous fish were low plated, whilst heterozygotes and *Eda*^*C*^ homozygotes were fully plated. We exposed the fish to the two lakes where their grandparents were caught by placing them into replicated semi-natural enclosures. Fish were individually marked which allowed us to calculate their growth over the course of the experiment, by measuring length at the start and end, and therefore examine any potential negative impacts of parasite infection. After 4 weeks of exposure, we measured the expression levels of a set of eight genes which give a general overview of the function of the immune system^50^, representing the innate pro-inflammatory response (*Il-1β* and *Tnfα*), Th1-type adaptive response (*Stat4* and *Tbet*), Th2-type adaptive response (*Stat6* and *Cmip*) and immune regulatory response (*Tgfβ* and *Foxp3*). This specific experimental design allows us to examine the link between *Eda* genotype and the function of the immune system under natural conditions, in two contrasting environments. This represents the first study, to our knowledge, to examine the link between a gene underlying adaptive phenotypic evolution and the key defences of an organism against potentially harmful pathogens and parasites, and adds to our growing understanding of the importance of unexpected additional effects through pleiotropy or linkage disequilibrium. We predicted that immune gene expression levels would differ with *Eda* haplotype block, and this effect would vary between the two exposure locations. We also predicted that fish with the wild type *Eda* haplotype block would be less susceptible to the saltwater parasite species, and that conversely fish with the derived *Eda* haplotype block would be less susceptible to the freshwater specific parasite. In addition to this, previous work in stickleback led us to predict that fish carrying the wild-type *Eda* haplotype block would grow faster than those with the freshwater *Eda* haplotype^16^,^22^.

## Results

### Eda genotype and immune gene expression

**Figure 1:**
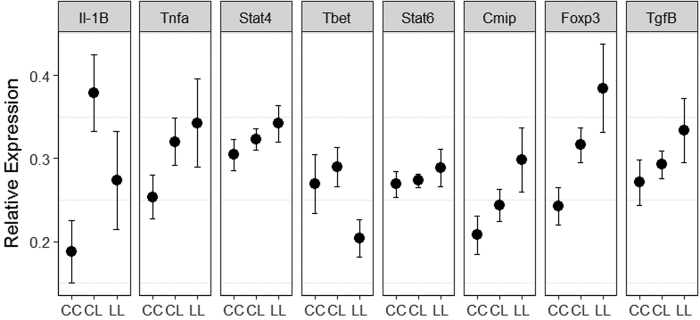
Relative expression levels (mean ± SE) of eight immune system genes which vary in multivariate space with *Eda* genotype, in fish exposed to natural conditions in two locations on North Uist, Scotland. *Eda* genotype determines armour plate phenotype, with two alternative alleles, denoted ‘C’ for the complete plated allele and ‘L’ for the low plated allele. All relative expression values were log_10_-transformed prior to analysis, and n = 46 for each response type.

We found differences in overall immune gene expression levels between fish with different *Eda* genotypes. Expression profiles differed between *Eda* genotypes (Fig. 1, MANOVA F_1,41_ = 2.68, p = 0.021), and between fish exposed in enclosures in different lakes (MANOVA F_1,41_ = 21.07, p < 0.001), but the effect of *Eda* did not change with enclosure location (MANOVA F_1,41_ = 1.28, p = 0.283).

**Figure 2:**
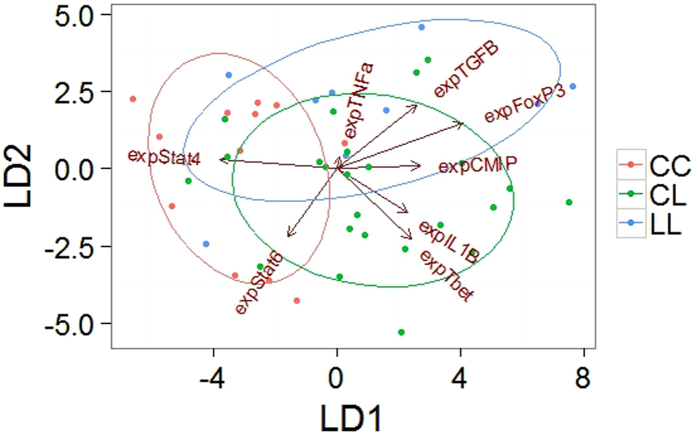
Individuals with different *Eda* genotypes were separated by multivariate immune gene expression profiles using linear discriminant analysis. *Eda* genotype determines armour plate phenotype, with two alternative alleles denoted ‘C’ for the complete plated allele and ‘L’ for the low plated allele. The length of the arrow for each gene indicates its relative contribution to the first and second linear discriminant axes, with ellipses indicating one standard deviation from the group mean. All relative expression values were log_10_-transformed prior to analysis, and n = 46.

Whilst we found a significant overall effect of *Eda* genotype on expression levels, only a single gene, *Foxp3* (ANOVA F_1,41_ = 9.14, p = 0.034) was significant in the individual gene comparisons (Fig. 1). All other genes did not differ significantly with *Eda* genotype (ANOVA’s, p values with false discovery rate adjustment for multiple tests): *Il-1β* F_1,41_ = 1.52, p = 0.225; *Tnfα* F_1,41_ = 3.04, p = 0.178; *Stat4* F_1,41_ = 1.92, p = 0.231; *Tbet* F_1,41_ = 2.52, p = 0.192; *Stat6* F_1,41_ = 0.87, p = 0.357; *Cmip* F_1,41_ = 4.39, p = 0.157; *Tgfβ* F_1,41_ = 3.78, p = 0.157). As the overall test of the effect of *Eda* was significant, we used linear discriminant analysis (LDA) to identify the best combination of genes by which to separate and visualise the three genotypes in multivariate space (Fig. 2). Linear discriminant 1 (LD1) accounted for 66% of between-group variance and largely separates high plated homozygous fish (genotype CC) from fish with a low plated allele (genotype CL or LL). LD1 was predominantly driven by expression of *Il-1β, Tbet, Cmip, Foxp3* and *Tgfβ* in one direction and opposing expression changes in *Stat4* and *Stat6*. Linear discriminant 2 (LD2) accounted for the remaining 34% of between-group variance, separating heterozygous fish (genotype CL) from low plated fish (genotype LL). The LD2 axis was driven by expression of *Foxp3* and *Tgfβ* with opposing changes in expression of *Il-1β, Tnfα* and *Stat6*.

Fish exposed in Obse had higher *Il-1β* (ANOVA F_1,41_ = 6.77, p = 0.021), *Tnfα* (ANOVA F_1,41_ = 13.40, p = 0.001), *Stat6* (ANOVA F_1,41_ = 15.83, p < 0.001) and *Tgfβ* (ANOVA F_1,41_ = 34.65, p < 0.001) expression levels than those in Chru, whilst expression of *Tbet* was higher in Chru than Obse (ANOVA F_1,41_ = 49.58, p < 0.001), possibly reflecting the different challenges faced in these two contrasting environments. Expression levels of *Stat4* (ANOVA F_1,41_ = 0.15, p = 0.806), *Cmip* (ANOVA F_1,41_ = 0.01, p = 0.974) and *Foxp3* (ANOVA F_1,41_ = 0.32, p = 0.767) did not differ with enclosure location (ANOVA p values adjusted with false discovery rate for multiple testing).

### Parasite infection dynamics

**Figure 3:**
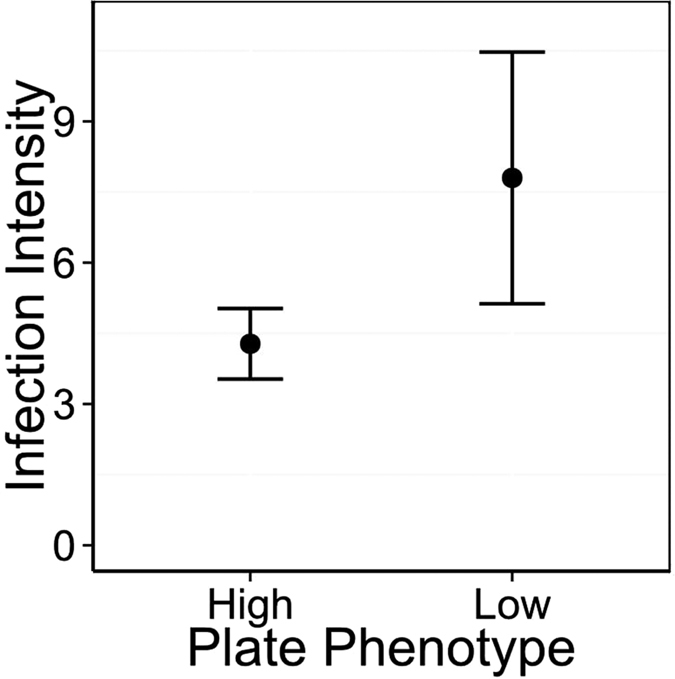
Individuals with the low plated phenotype were infected with a higher number of *G. arcuatus* parasites than individuals with the high plated phenotype. Infection intensity is the mean number of parasites found on infected individuals ( ± SE, n = 86), after 4 weeks of exposure to natural conditions in Obse.

Fish were infected with *G. arcuatus* in Obse (prevalence 67.6%) and *D. gasterostei* in Chru (prevalence 5.7%), with prevalence of *D. gasterostei* too low for further analysis. Low plated fish were infected with a higher number of *G. arcuatus* than high plated fish (Fig. 3, χ^2^ = 6.04, p = 0.014) with a mean parasite burden of 2.96 ( ± 0.65) on high plated fish and 4.88 ( ± 2.14) on low plated fish.

### Eda genotype and growth

**Figure 4:**
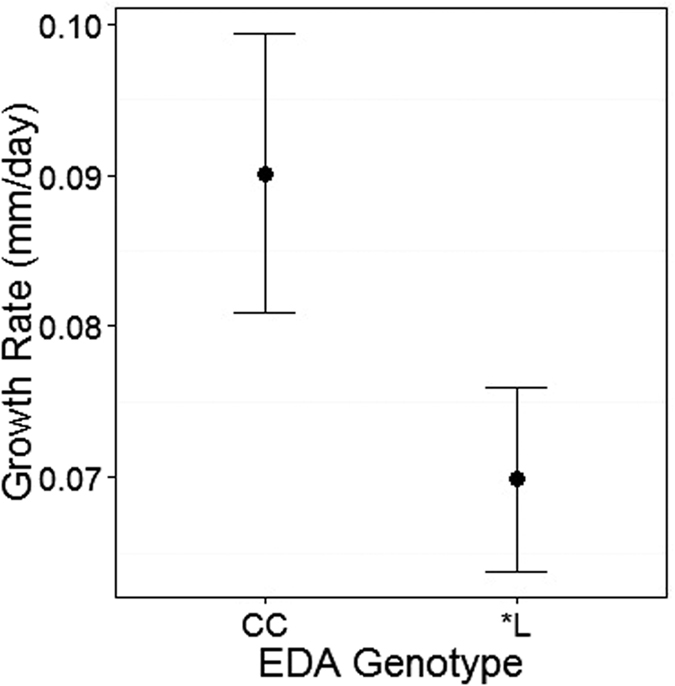
Growth rates (Mean ± SE, n = 46) of experimental fish, exposed to natural conditions, differed with Eda genotype. *Eda* genotype determines armour plate phenotype, with two alternative alleles denoted ‘C’ for the complete plated allele, ‘L’ for the low plated allele, or ‘*’ where individuals can have either the C or L allele.

The growth rate of each individual was calculated as increase in length per day over the course of the experiment. Fish with two complete *Eda* alleles (genotype CC) grew faster than fish with a low plated allele (genotype CL or LL) irrespective of enclosure location (Fig. 4, Wald F_1,38_ = 6.95, p = 0.012). The slow growth of LL fish in Obse was particularly evident on visual examination of the data, but the enclosure location by *Eda* genotype interaction term was non-significant and so not included in the final model.

## Discussion

In this study, we employed a common garden based approach, exposing laboratory raised fish to natural conditions, to show an association between the *Eda* haplotype block and the expression levels of key immune system genes. By using F2 hybrids, we were able to create fish that varied in *Eda* genotype whilst controlling for background genetic variation and maternal effects. Evolutionary genetic theory predicts that loci which contribute to differences in local adaption between populations are themselves likely to be pleiotropic, or will cluster with similarly adaptive loci in tight physical linkage to form ‘supergenes’^4^,^6^. Previous work has shown *Eda* genotypes (haplotypes) to relate to growth, behaviour, pigmentation, and sensory system development^14^,^15^,^16^, and here we demonstrate an additional link with the immune system. Overall gene expression levels, used here as an indicator of immune system activity, differed between fish with different *Eda* genotypes, above any variation seen between the environments in which these individuals were exposed. As fish were laboratory raised with no previous exposure to stressful conditions, the patterns observed likely relate to *Eda* genotype, either directly or through the action of linked genes.

A number of possible mechanisms may explain the observed association between the *Eda* haplotype block and immune gene expression levels. Differences could represent pleiotropic effects of *Eda* itself, possibly through the regulatory change which dictates plate number during development^51^, or *Eda* genotype, through plate phenotype, could result in the differing parasite burdens observed, which in turn result in expression differences. Alternatively, the pattern could arise from the linkage of *Eda* with other genes with roles in immune function pathways. The block of tight linkage surrounding *Eda* contains a number of genes, including *Baff, Garp*, and *Dusp1*^17^,^18^,^19^, which have important roles in the function of the immune system. *Garp* promotes *Tgfβ* expression and regulates the T cell response^38^,^39^,^40^, although we did not see any direct changes in *Tgfβ* expression levels*. Dusp1* controls inflammation levels^41^,^42^, and we observed a strong but non-significant difference in expression of *Il-1β*, a major driver of the inflammatory response, between fish carrying alternative *Eda* haplotype combinations. Changes in expression levels of genes in the same pathways as genes in linkage with *Eda* demonstrate the strong possibility of a link between immune function and this genomic region, and previous studies have disentangled the effects of *Eda* itself versus those of genes in tight linkage^52^. In this instance, we employ assays previously used in stickleback immunological studies which measure the overall immune response, rather than the expression of the specific genes within the *Eda* haplotype region. By using this approach it is possible to see whether there is a relationship between the *Eda* haplotype region and the wider immune response, although we may miss changes occurring in concert with *Eda* if they act through other immune response pathways. Direct measurement of the expression of the genes in close linkage with *Eda* would address this issue, and along with examination of genomic sequence, could establish the underlying cause of immune changes and whether these occur in concert with *Eda* during adaptation.

Differences in expression levels between lakes would be expected, based on previous studies employing these assays^50^,^53^. Here we see significant expression differences between exposure locations; as a common garden approach was used we know the differences in this instance must be a result of the environment experienced by an individual, as opposed to individual variation in life history or past experience. Even with these environmental differences, we are still able to see a link between *Eda* haplotype and gene expression levels. In Obse, where infection with *G. arcuatus* is common, we found that low plated fish were infected with a higher number of parasites than high plated fish. This roughly two-fold difference in abundance between plate morphs is similar to the difference between abundance observed between MHC genotypes in a previous study^34^. As *G. arcuatus* attaches to the external surface of a fish, it could be easier for parasites to attach to fish that do not have plates. This difference in parasite burden could then drive the differences in immune expression levels seen with *Eda* genotype. Alternatively, low plated fish may be unable to mount a successful response to infection, or their underlying immune expression may make them more susceptible to infection, as the L allele is not adaptive in the saltwater environment where this parasite is common. Whilst we would expect the opposite to be true for *D. gasterostei*, it was not possible to test this as infection prevalence was too low.

An individual’s *Eda* genotype was associated with growth, with CC genotype fish growing faster than fish with an L allele. Fish carrying the low allele have previously been found to grow faster as juveniles, but this advantage appears to disappear in adult fish^16^,^22^. In the wild, fish from Chru are typically smaller than those in Obse. The lower growth of fish with the L allele from Chru could thus be the result of this difference in adult size, if adult size is also determined by *Eda* or linked genes. Although non-significant in the model, the slow growth of LL fish was particularly evident in Obse, where it could be due to the non-adaptedness of the L allele or a result of the higher *G. arcuatus* infection levels observed.

The stickleback-*Eda* system represents one of the best studied examples of an adaptive phenotypic change and its underlying genetic control, yet the full range of pleiotropic and linked effects of this adaptation have yet to be discovered. Previous studies on *Eda* have shown pleiotropic effects on growth, behaviour and sensory system development^14^,^15^,^16^, and here we found that *Eda* was associated with the expression of genes giving an overall indication of immune system function, along with growth and parasite infection levels. Parasites have the potential to drive adaptive evolution^54^ if they have a negative impact on fitness and genetic variation in resistance resulting in uneven parasite distributions^55^. Parasites may represent an additional selective pressure on the *Eda* locus, either as a direct result of pleiotropy, or through selection on linked genes involved in the immune response. Further work is required to better elucidate the role and mechanisms underlying the observed variation in immune gene expression levels. We demonstrate a previously undescribed link between a widely studied gene underlying adaptation to new environments and the function of a key organismal process, as well as hypothesising the mechanisms which may underlie this relationship. This study underlines the potential of genomic regions containing physically linked genes, including genes of large effect involved in adaptation, to affect additional processes with potentially important adaptive consequences.

## Methods

All work involving animals was approved by the University of Nottingham ethics committee, and performed under UK Home Office licence (PPL-40/3486) in accordance with UK Home Office regulations.

### Fish crossing

We created eight F1 families in May 2011 by crossing individuals from Loch Ob nan Stearnain (‘Obse’, 57°36′9″N; 7°10′19″W), a saltwater lagoon containing fully plated resident fish, with individuals from Loch à Chadha Ruaidh (‘Chru’, 57°35′38″; 7°11′51″W), a freshwater population with low armour plating, both located on North Uist, Scotland. Each population contributed an even number of males and females, and we performed crosses following the procedure of De Roij *et. al.*^45^. Fertilized eggs were transported to aquaria at the University of Nottingham, with families split between multiple tanks after hatching to ensure all fish were maintained at the same density. Fish were kept in a temperature controlled room, with a lighting regime mirroring the natural photoperiod on North Uist. Four F2 families were then created in May 2012 by crossing F1 fish from unrelated families, following the same method.

### Experimental exposure

We randomly assigned five fish from each of the first three families, and four from the fourth family, to each of ten treatment groups, giving 19 fish per group and 190 fish in total. Five groups were exposed to semi-natural conditions in Chru, and five in Obse. Prior to exposure, we tagged all fish with visible implant elastomer tags (VIE, Northwest Marine Technology) so that individuals could be identified within each treatment group. Tagging was performed two months prior to the start of the experiment, to allow fish sufficient time to recover and to ensure that their immune response was not affected by the tagging procedure^56^. The length of each individual was recorded at the time of tagging. As Obse is a saltwater lagoon, fish assigned to an Obse enclosure were gradually acclimatised to saltwater conditions two months prior to the start of the experiment (from 2 parts per thousand [ppt] salt to 20 ppt), and all fish were kept on a daylight and temperature regime mirroring that on North Uist.

Fish were transported to North Uist in May 2013. We constructed ten enclosures using 6 mm square mesh, with each enclosure being cylindrical in shape, 90 cm long and 68 cm in diameter, giving a total volume of 330 cm^3^. This mesh size was selected to contain fish within the enclosures whilst still allowing the free flow of water and suspended materials. All fish from each treatment group were added to a single enclosure.

After 4 weeks, we removed all fish from a single, randomly selected, enclosure at a time. Fish were transported to the laboratory in darkened boxes containing fresh loch water, with air supplied from a battery powered pump. We processed all fish in a haphazard order within two hours of collection. No correlation was found between immune gene expression levels and sampling order, indicating that holding time had no significant effect on our immune system measures (see Supplementary Results).

Fish were euthanized by overdose of MS222 (Sigma-Aldrich) followed by destruction of the brain, in accordance with UK Home Office regulations. Length was measured, and the spleen, an immunologically important tissue in fish^57^, was removed and immediately placed in RNAlater (Life Technologies). Each fish was identified using its unique VIE tag, and we counted macroparasites under a dissection microscope. Each individual’s plate phenotype was determined visually. We recorded sex and reproductive status by examination of the gonads. Fish were split into three reproductive status categories: no apparent investment in reproduction, investment in attaining reproductive condition, or ready to breed. A sample of fin tissue was removed and stored in 100% ethanol for *Eda* genotyping.

### Gene expression quantification

The full RNA extraction, reverse transcription, and qPCR methods used are detailed in the Supplementary Methods. From those samples passing strict quality control steps during extraction and reverse transcription, we randomly selected a subset of 46 (giving one full qPCR plate) for inclusion in the gene expression analysis study, with 26 samples from fish exposed in Chru (*Eda* genotype n: CC = 7, CL = 14, LL = 5) and 19 from Obse (*Eda* genotype n: CC = 5, CL = 10, LL = 5). We measured the expression levels of eight genes of interest, along with two reference genes (*HPRT1* and *TBP*) selected for their stability in our experimental samples (see Supplementary Methods). Genes of interest were interleukin 1 beta (*Il-1β)*, tumour necrosis factor alpha (*TNFα)*, signal transducer and activator of transcription 4 (*Stat4)*, T box transcription factor 21 (*Tbet)*, signal transducer and activator of transcription 6 (*Stat6)*, c-Maf inducing protein (*Cmip)*, forkhead box p3 (*Foxp3)* and transforming growth factor beta (*Tgfβ)*. These genes have previously been used to examine the immune response of stickleback, and were selected to give an overview of the function of the immune system at the time of sampling (For full details, see ref. 50). Relative expression values were calculated using the ΔΔCq method^58^, adjusted for the amplification efficiencies of each primer pair and standardised against the geometric mean Cq of the two reference genes for each sample^59^.

### Eda genotyping

Individuals selected for expression analysis were genotyped using *Stn382* primers, which flank an indel polymorphism in intron 1 of the *Eda* gene^17^. We extracted DNA from fin tissue using DirectPCR Lysis Reagent for Mouse Tails (Viagen Biotech), by incubating for 12 hours at 55 °C followed by 45 minutes at 85 °C. PCR reactions were performed in a 20 μl total reaction volume containing 10 μl BioMix Red Mastermix (Bioline), 6μ H_2_O, 1 μl forward and 1 μl reverse primer at 10 μM, and 2 μl template DNA, with an initial step of 94 °C for 4 minutes, followed by 35 cycles of 94 °C for 15 seconds, 60 °C for 15 seconds and 72 °C for 15 seconds, and a final extension of 72 °C for 4 minutes. We visualised PCR product on a 2% Agarose gel containing Ethidium Bromide, with the complete plated allele giving a product 218 bp in length, and the low plated allele 150 bp in length. Both wild type homozygous (*Eda* genotype CC) and heterozygous (genotype CL) fish were found to be high plated, whilst derived homozygous fish (genotype LL) were low plated.

### Data analysis

All gene expression data was log_10_(x + 1) transformed prior to analysis, due to the inherently skewed distribution of relative expression data. We performed all analyses in R v.3.2.2^ ^60.

#### EDA genotype and immune gene expression

To examine whether there was a relationship between *Eda* genotype and gene expression levels, we fitted a MANOVA with log_10_-transformed relative expression values as the response. Enclosure location, the number of *Eda* L alleles carried (to represent *Eda* genotype) and their interaction were fitted as factors, with overall differences assessed using the Pillai method. For significant terms, the effect on each immune response type was then examined separately using ANOVA, with the false discovery rate (FDR) method applied to control for multiple comparisons^61^. To visualise the overall differentiation of multivariate immune gene expression profiles between *Eda* genotypes, and identify which genes best separate these groups in multivariate space, we performed a linear discriminant analysis using the ‘lda’ function of the ‘MASS’ package.

#### Parasite infection dynamics

Infection levels of *D. gasterostei* were too low to allow any analysis. The number of *G. arcuatus* on individuals exposed in Obse was compared between fish differing in armour plating. We used plate phenotype rather than genotype in this instance as phenotype data was available for a larger number of individuals. A generalised linear model was fitted with plate level as a factor (high or low plated) and infection intensity (parasite burden) as the response, with a Poisson error structure. Significance was calculated using likelihood ratio tests.

#### Growth variation

As individual fish were marked, we could examine whether *Eda* genotype related to growth over the course of the experiment. Individual growth rates were calculated as increase in length per day (mm/day). A general linear model was fitted with growth rate as the response. Maximal models were fitted with enclosure location (2 levels), sex (2 levels), reproductive status (3 levels) and *Eda* genotype (3 levels) as factors, along with the interaction of each factor with location. Non-significant terms were sequentially dropped to give a minimum adequate model, and the significance of remaining components was determined by Wald F-tests.

## Additional Information

**How to cite this article**: Robertson, S. *et al*. Eda haplotypes in three-spined stickleback are associated with variation in immune gene expression. *Sci. Rep.* **7**, 42677; doi: 10.1038/srep42677 (2017).

**Publisher's note:** Springer Nature remains neutral with regard to jurisdictional claims in published maps and institutional affiliations.

## Supplementary Material

Supplementary Materials
