# Supplementary material for: Eda haplotypes in three-spined stickleback are associated with variation in immune gene expression
Source: Sci Rep. 2017 Feb 14;7:42677. doi: 10.1038/srep42677 (PMC5307360; doi:10.1038/srep42677)
Supplement: Supplementary Materials [file srep42677-s1.pdf]

Supplementary materials for ‘Eda haplotypes in three-spined stickleback are associated with immune gene expression’

Shaun Robertson, Janette E. Bradley and Andrew D.C. MacColl

Supplementary Results 1

Sampling Order and Gene Expression Levels. Due to the rapid and responsive nature of the immune system, we checked whether the time fish were held before processing and the order in which they were processed affected the expression levels of our genes of interest. We calculated the correlation between sampling order and each of the log-transformed immune gene expression levels, using the 'corr.test' function of the 'psych' package in R v.3.1.2 with the false discovery rate (fdr) correction applied for multiple comparisons.

No significant correlations were found between sampling order and *IL-1β* (r^2=0.09, p=0.64), *TNFα* (r2=-0.23, p=0.25), *Stat4* (r^2=-0.26, p=0.19), *Tbet* (r^2=0.18, p=0.37), *Stat6* (r^2=-0.11, p=0.60), *CMIP* (r^2=-0.21, p=0.31), *FoxP3* (r^2=-0.14, p=0.51), and *TGFβ* (r^2=-0.01, p=0.98). The order in which fish were sampled, with later fish being held for longer before sample collection, had no detectable effect on the expression levels of the genes measured.

Supplementary Methods 1

Sample Preparation. We extracted RNA from Spleens using the GeneJET RNA purification kit (Thermo Scientific) according to the manufacturers' standard protocol. Purity and concentration of RNA was assessed on a NanoDrop 1000 spectrophotometer (Thermo Scientific), with a desired 260/280 absorbance ratio >1.80. RNA integrity was assessed by incubating 5µl of each sample at 65°C for 10 minutes, followed by visualisation on a 2% Agarose gel stained with Ethidium Bromide. All samples were DNase treated using Precision DNase (Primer Design), following the manufacturers' protocol. Reverse transcription reactions were performed on approximately 1.5µg of total RNA using the nanoScript2 RT kit (Primer Design), with a combination of random nanomer and oligo-dT priming, following the manufacturers' protocol. We included periodic no-enzyme controls, which indicated that genomic DNA contamination was negligible. All cDNA samples were diluted 1:10 with nuclease free water before further use.

Reaction Conditions. qPCR reactions were performed in 10µl total volumes, containing 5µl of PrecisionFAST mastermix with SYBR green (Primer Design), 0.25µl of each primer, 2µl of template cDNA and 2.5µl of H_2O in 96-well optical PCR plates with optical seals (StarLab). Reactions were performed in an ABI 7500 FAST real-time thermocycler (Applied Biosystems) with an initial step of 95°C for 20 seconds, followed by 45 cycles of 95°C for 3 seconds and 60°C for 30 seconds. All runs included a post-PCR melt curve analysis.

Supplementary Methods 2

Accurate normalization is essential for the production of reliable data from qPCR experiments, with the optimal reference genes being specific to a particular set of experimental conditions[1]. In order to select the most appropriate reference genes for this study, we performed a geNorm analysis on 15 randomly selected samples (7 from Chru and 8 from Obse) with six candidate reference genes (B2M, GAPDH, RPL13A, HPRT1, TBP and TOP1), using a custom stickleback geNorm kit for SYBR green (Primer Design) following the manufacturers standard protocol. Analysis of the stability of expression was performed in qbase+ (Biogazelle) with HPRT1 and TBP identified as the most stable combination of reference genes.

Supplementary References

1 Dheda, K. *et al.* The implications of using an inappropriate reference gene for real-time reverse transcription PCR data normalization. *Analytical biochemistry* 344, 141-143, doi:10.1016/j.ab.2005.05.022 (2005).
